# QSPR modelling of PPI drugs using hybrid topological indices

**DOI:** 10.3389/fchem.2026.1896796

**Published:** 2026-08-11

**Authors:** L. Monisha, R. Jayagopal

**Affiliations:** Department of Mathematics, School of Advanced Sciences, Vellore Institute of Technology, Chennai, India

**Keywords:** edge partition, hybrid topological indices, molecular structure, PPIs, QSPR analysis, regression model

## Abstract

PPIs are one of the most often prescribed drug groups for ailments involving high stomach acid production. PPIs permanently suppress the H+/K + -ATPase enzyme in gastric parietal cells, blocking acid secretion. Topological indices are quantitative analysers that are used to define the structural attributes of molecules which transmit topological information such as adjacency, branching and connectivity to numerical values. In this paper, we explore the chemical significance of degree-based hybrid topological indices for proton pump inhibitors (PPIs) and evaluate their correlation with the physicochemical properties of PPIs. Our goal is to utilize Quantitative Structure Property Relationship analysis to identify correlations between structural characteristics and physicochemical properties. This approach underscores the value of topological indices in evaluating the properties of PPIs. This analysis helps to comprehend structural activity correlations in domains such as drug design and material research by providing insights into molecular behaviour.

## 1 Introduction

Proton Pump Inhibitors (PPIs) are an exceptionally effective class of drugs that are currently extensively used to treat various types of acid related gastrointestinal diseases. The primary mechanism by which PPIs work is by blocking the hydrogen-potassium ATPase enzyme complex, which is also known as the proton pump, located within the stomach’s gastric parietal cells. PPIs are more potent compared to various acid-suppressing drugs like H2-receptor antagonists due to the fact their proton pump inhibition remains strong and long-lasting. A decline in acid production aids in controlling underlying symptoms and mending of damaged tissue triggered by conditions such as GERD, peptic ulcers and Zollinger-Ellison syndrome. GERD is a chronic disorder characterised by the reflux of stomach acid into the oesophagus, resulting in symptoms such as heartburn and food regurgitation. PPIs are vital for dealing with acid hypersecretion in individuals experiencing Zollinger-Ellison syndrome, a rare disorder characterised by the growth of tumours that enhance acid production, resulting in severe peptic ulcers. PPIs have been associated with diminished rates of medical conditions like oesophageal structure, Barrett’s oesophagus and gastrointestinal haemorrhage. Persistent intake of PPIs might hinder the absorption of nutrients, such as calcium, magnesium, and vitamin B12, which might result in deficits. Magnesium insufficiency can lead to muscle spasms, arrhythmias and seizures, whereas calcium deficiency weakens bones and increases the risk of fractures. Vitamin B12 deficiency can result in anaemia and neurological problems. Research suggests that long term PPI usage may be associated with an increased risk of bone fractures in hip, wrist and spine due to reduced calcium absorption. It is necessary to routinely evaluate and track individuals undergoing PPI medication in order to reduce these hazards. Nutritional modifications and managing weight are instances of modifications to lifestyle that may encourage enhancement of PPI therapy. Many researchers have extensively investigated proton pump inhibitors (Agarwal et al., 2013; Moo Shin and Sachs, 2008; Sachs et al., 2006; Strand et al., 2017; Vanderhoff and Tahboub, 2002). Although PPIs offer significant therapeutic benefits, their long-term use is associated with several adverse effects. Therefore, it is important to analyze their molecular properties to aid in the design of safer drugs with minimal side effects. In this regard, topological indices and QSPR analysis provide an effective approach for studying PPIs.

Chemical graph theory is a branch of mathematical chemistry that deals with chemical graphs that represent chemical systems. In theoretical chemistry, drugs are modeled as molecular networks, with each vertex representing an atom and each edge indicating a relationship between two atoms. Topological indices are numerical parameters derived from the molecular graph of a chemical compound, providing valuable insights into its structure and properties without the need for detailed molecular modelling. Degree-based indices play a fundamental role by quantifying the connectivity of atoms within the molecule. Degree-based hybrid topological indices constitute a class of descriptors obtained by combining two or more degree-related graph characteristics within a single mathematical expression. Unlike classical degree-based indices, which typically quantify a single aspect of vertex connectivity, hybrid descriptors simultaneously incorporate complementary degree information through nonlinear combinations such as products, ratios, or weighted interactions. Consequently, they provide a richer mathematical characterization of the local structural environment of a molecular graph while preserving the computational simplicity of degree-based descriptors. Their ability to encode multiple connectivity features has motivated their successful application in QSPR and QSAR investigations. The present work considers twelve representative hybrid degree-based topological indices that have recently attracted attention in mathematical chemistry. Although these indices are all degree-based, they differ in their mathematical formulations and emphasize different aspects of molecular connectivity. The objective of this study is to investigate their relative effectiveness in modelling the physicochemical properties of proton pump inhibitor drugs and to identify those descriptors that exhibit the strongest predictive capability for the molecular dataset under consideration.

Recent years have witnessed substantial progress in the application of topological descriptors and QSPR modelling for pharmaceutical compounds. Several studies have explored antituberculosis drugs (Adnan et al., 2022), asthma medications using neighborhood degree-based descriptors (Balasubramaniyan and Chidambaram, 2023), antiviral drugs including Molnupiravir for COVID-19 treatment (Das et al., 2023), and breast cancer therapeutics (Haq Bokhary et al., 2022). QSPR investigations have also been reported for cardiovascular drugs (Bashir Farooq et al., 2022), NSAID medications (Rosalind Mary Gnanaraj et al., 2023), heart attack treatments (Abdul et al., 2023), calcium channel-blocking cardiac drugs (Hasani et al., 2024) and anti-malaria compounds (Zhang et al., 2023).

Furthermore, topological approaches have been employed to analyze pulmonary cancer drugs, Parkinson’s disease medications, Lyme disease drugs, Hepatitis prescriptions, blood cancer and skin cancer therapeutics (Ahmed et al., 2025; Arockiaraj et al., 2025a; Arockiaraj et al., 2025b; Chen et al., 2025; Hayat and Wazzan, 2025; Huang et al., 2023a; Huang et al., 2023b; Jeni Godlin and Radha, 2026; Mahboob et al., 2023; Mahboob et al., 2024; Paul et al., 2025; Qin et al., 2025; Zhang et al., 2024). In addition, several graph families, chemical structures, isomers, and dendrimers have been extensively examined through topological indices and predictive modelling (Arockiaraj et al., 2019; Chu et al., 2022; Gurunathan et al., 2023; Huang et al., 2022; Kulli, 2018; Mohammed et al., 2020; Mondal et al., 2021; Paul et al., 2023; Raza et al., 2023a; Raza et al., 2023b; Shanmukha et al., 2021; Zhang et al., 2022). These developments demonstrate the growing importance of topological descriptors in modern cheminformatics and drug design.

Recent advances in chemical graph theory have expanded the range of topological descriptors employed in QSPR and QSAR investigations. In addition to traditional degree-based indices resistance based descriptors and distance-based descriptors such as the Kirchhoff index (Shoaib and Sardar, 2025) have attracted considerable attention due to their ability to capture global structural characteristics of molecular graphs and provide complementary information for property prediction. Furthermore, reverse degree-based descriptors (Mudassar Hassan et al., 2026) have demonstrated promising applications in the study of molecular and nanostructural systems by offering alternative perspectives on structural complexity. Despite these developments, degree-based topological indices continue to remain widely used because of their mathematical simplicity, computational efficiency and straightforward structural interpretation. Moreover, hybrid descriptors have shown significant effectiveness in establishing structure property relationships for diverse classes of chemical compounds. Motivated by these advantages, the present study focuses on degree-based hybrid topological descriptors for the QSPR analysis of proton pump inhibitor compounds.

The integration of topological descriptors with machine learning algorithms has significantly expanded the scope of QSPR and QSAR modelling by enabling the development of more flexible and accurate predictive models (Shi et al., 2025; Shoaib Sardar et al., 2025). While such approaches have demonstrated promising predictive capabilities, regression models based on topological descriptors continue to offer advantages in terms of simplicity, computational efficiency, and interpretability. Consequently, the present study provides a foundation for future investigations that incorporate the proposed hybrid topological descriptors into machine-learning-based QSPR/QSAR frameworks.

**FIGURE 1:**
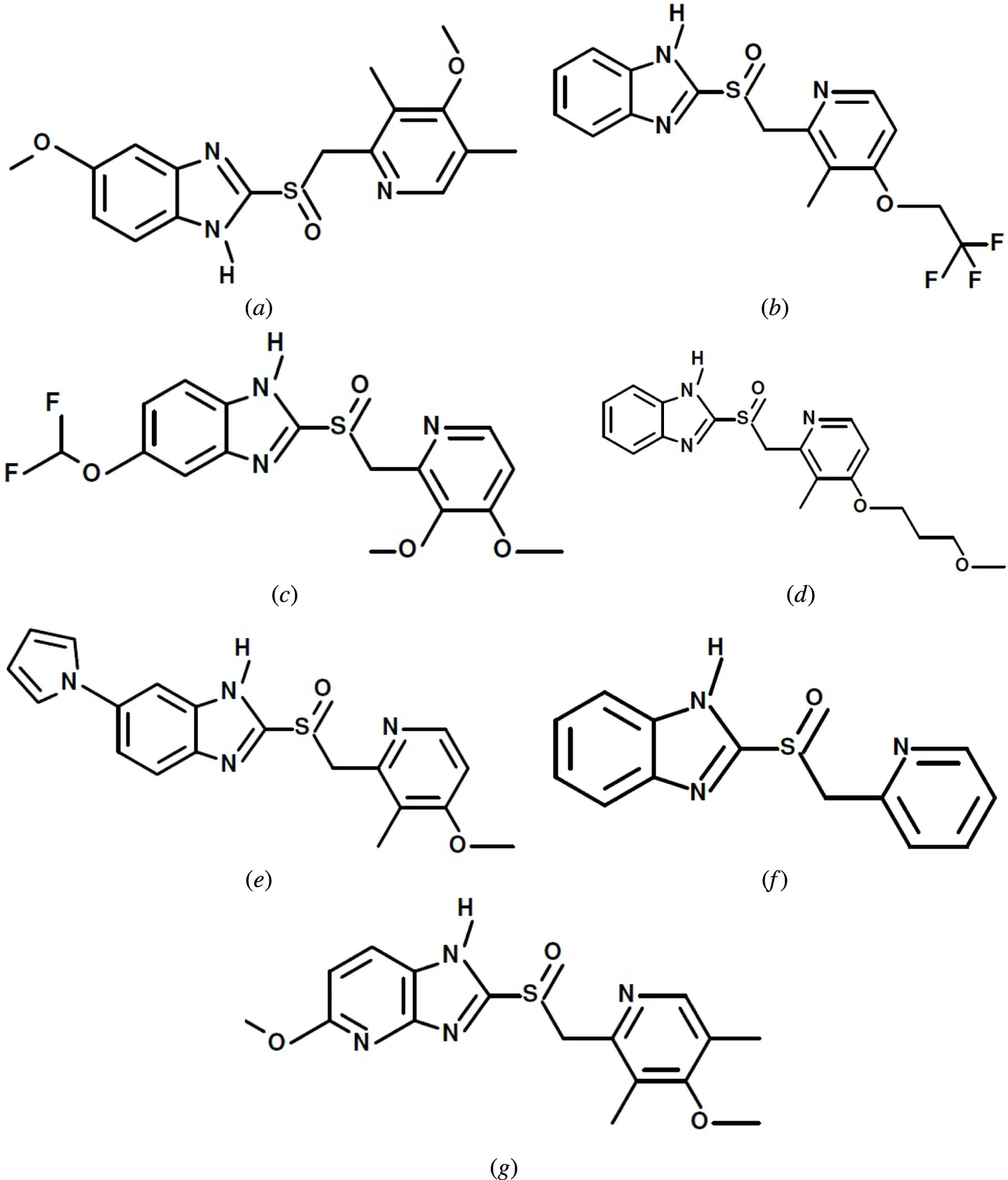
PPI molecules **(a)** Omeprazole; **(b)** Lansoprazole; **(c)** Pantoprazole; **(d)** Rabeprazole; **(e)** Ilaprazole; **(f)** Timoprazole and **(g)** Tenatoprazole.

In this study PPIs, namely, Omeprazole, Lansoprazole, Pantoprazole, Rabeprazole, Ilaprazole, Timoprazole and Tenatoprazole (Figure 1) are considered. Several regression models are employed in QSPR analysis to investigate the relationship between the hybrid topological indices and the physicochemical properties of PPI drugs.

## 2 Methodology

Let G be a simple connected graph with vertex set V(G) and edge set E(G) . The degree of a vertex v∈V(G) is denoted by $d_{v}$ . Two vertices u,v∈V(G) are adjacent whenever uv∈E(G) (Douglas, 2001). Vertex degree is a fundamental structural descriptor in chemical graph theory (Hayat et al., 2018; Hayat et al., 2019). This work focuses on hybrid degree-based topological indices which extend standard degree-based descriptors by integrating multiple structural components into a unified framework. Unlike conventional indices that rely on a single degree-based measure, hybrid indices combine different mathematical formulations to capture more intricate structural information of molecular graphs. This integration enhances their predictive capability and makes them more suitable for QSPR modelling of complex chemical compounds. Hybrid indices which provide more comprehensive characterization of molecular topology by incorporating diverse degree related interactions within the graph structure. By incorporating both vertex connectivity and edge interaction information hybrid descriptors provide a broader representation of molecular topology than indices derived from a single degree parameter. Consequently, they are capable of reflecting multiple degree-related structural characteristics within a unified mathematical framework which makes them suitable for structure property investigations. Owing to these advantages hybrid degree-based indices have emerged as effective tools in contemporary QSPR and QSAR investigations.

### 2.1 Hybrid Zagreb topological indices

The hybrid Zagreb topological indices (Arockiaraj et al., 2023) considered in this study are given below:

- Bilinear Zagreb Index

$$BM\left(G\right)=\underset{u\in V\left(G\right)}{\sum }d_{u}^{2}+\underset{uv\in E\left(G\right)}{\sum }d_{u}d_{v}=\underset{uv\in E\left(G\right)}{\sum }\left(d_{u}+d_{v}+d_{u}d_{v}\right)$$

- Triangular Zagreb Index

$$TM\left(G\right)=\underset{u\in V\left(G\right)}{\sum }d_{u}^{3}+\underset{uv\in E\left(G\right)}{\sum }d_{u}d_{v}=\underset{uv\in E\left(G\right)}{\sum }\left(d_{u}^{2}+d_{v}^{2}+d_{u}d_{v}\right)$$

- Geometric–Harmonic Index

$$GH\left(G\right)=\underset{uv\in E\left(G\right)}{\sum }\frac{\sqrt{d_{u}d_{v}}\left(d_{u}+d_{v}\right)}{2}$$

- Geometric–Bilinear Zagreb Index

$$GBM\left(G\right)=\underset{uv\in E\left(G\right)}{\sum }\frac{\sqrt{d_{u}d_{v}}}{d_{u}+d_{v}+d_{u}d_{v}}$$

- Geometric–Triangular Zagreb Index

$$GTM\left(G\right)=\underset{uv\in E\left(G\right)}{\sum }\frac{\sqrt{d_{u}d_{v}}}{d_{u}^{2}+d_{v}^{2}+d_{u}d_{v}}$$

- Harmonic–Geometric Index

$$HG\left(G\right)=\underset{uv\in E\left(G\right)}{\sum }\frac{2}{\sqrt{d_{u}d_{v}}\left(d_{u}+d_{v}\right)}$$

- Harmonic–Bilinear Zagreb Index

$$HBM\left(G\right)=\underset{uv\in E\left(G\right)}{\sum }\frac{2}{\left(d_{u}+d_{v}+d_{u}d_{v}\right)\left(d_{u}+d_{v}\right)}$$

- Harmonic–Triangular Zagreb Index

$$HTM\left(G\right)=\underset{uv\in E\left(G\right)}{\sum }\frac{2}{\left(d_{u}^{2}+d_{v}^{2}+d_{u}d_{v}\right)\left(d_{u}+d_{v}\right)}$$

- Bilinear–Geometric Index

$$BMG\left(G\right)=\underset{uv\in E\left(G\right)}{\sum }\frac{d_{u}+d_{v}+d_{u}d_{v}}{\sqrt{d_{u}d_{v}}}$$

- Bilinear–Harmonic Index

$$BMH\left(G\right)=\underset{uv\in E\left(G\right)}{\sum }\frac{\left(d_{u}+d_{v}+d_{u}d_{v}\right)\left(d_{u}+d_{v}\right)}{2}$$

- Triangular–Geometric Index

$$TMG\left(G\right)=\underset{uv\in E\left(G\right)}{\sum }\frac{d_{u}^{2}+d_{v}^{2}+d_{u}d_{v}}{\sqrt{d_{u}d_{v}}}$$

- Triangular–Harmonic Index

$$TMH\left(G\right)=\underset{uv\in E\left(G\right)}{\sum }\frac{\left(d_{u}^{2}+d_{v}^{2}+d_{u}d_{v}\right)\left(d_{u}+d_{v}\right)}{2}$$

## 3 Approach and evaluation

In order to analyze the twelve degree-based hybrid topological indices that are specified for modelling the seven common physical properties of the PPI drugs such as Omeprazole, Lansoprazole, Pantoprazole, Rabeprazole, Ilaprazole, Timoprazole and Tenatoprazole the edge partitioning technique has been impelmented to compute the degrees of all two-dimensional structures. In the molecular graph representation, atoms are considered as vertices and chemical bonds as edges. Following the standard approach in chemical graph theory, multiple bonds were treated as single edges while constructing the molecular graphs. For each molecular graph, the degree of a vertex was defined as the number of edges incident to that vertex. Subsequently, the edges were partitioned according to the degree pairs $\left(d_{u},d_{v}\right)$ of their end vertices, where $d_{u}$ and $d_{v}$ denote the degrees of the two incident vertices. The frequency of each degree pair was then determined by examining every edge in the molecular graph exactly once, and the resulting frequencies constitute the edge partitions reported in Table 1. These edge partitions serve as the basis for the computation of all degree-based hybrid topological indices considered in the present study. For example, an edge joining a vertex of degree 2 and a vertex of degree 3 contributes one count to the (2, 3) edge partition. Repeating this procedure for all edges of the molecular graph yields the complete edge partition. The chemical structures of the seven PPI drugs are shown in Figure 1, and Table 1 lists the corresponding degree-based edge partitions. We analyze the degree-based hybrid topological indices in relation to the physicochemical properties of these drugs listed in Table 2. The properties considered include boiling point (BP), molar refractivity (MR), molar volume (MV), molecular weight (MW), complexity (COM), polarizability (POL), and LogP. All physicochemical data were obtained from https://www.chemspider.com and https://pubchem.ncbi.nlm.nih.gov.

**TABLE 1:** Edge partitions of PPI compounds.

Drug	E(1,2)	E(1,3)	E(1,4)	E(2,3)	E(2,4)	E(2,2)	E(3,3)
Omeprazole	2	2	3	11	1	6	5
Lansoprazole	-	2	-	11	1	6	4
Pantoprazole	2	3	-	16	-	3	4
Rabeprazole	1	2	-	11	-	9	4
Ilaprazole	1	3	-	15	-	6	5
Timoprazole	-	1	-	10	-	7	2
Tenatoprazole	2	3	-	14	-	2	4

**TABLE 2:** PPIs and their physiochemical properties.

Drug	BP	MR	MV	MW	COM	POL	LogP
Omeprazole	600.0	94.0	251.90	345.40	453	37.3	2.23
Lansoprazole	555.8	88.0	245.90	369.40	480	34.9	2.84
Pantoprazole	586.9	91.4	252.70	383.40	490	36.2	2.11
Rabeprazole	603.9	98.7	269.00	359.40	440	39.1	2.00
Ilaprazole	651.0	102.8	263.10	366.40	502	40.8	3.04
Timoprazole	540.3	72.0	176.50	257.31	313	28.6	1.06
Tenatoprazole	591.5	91.8	244.80	346.40	455	36.4	1.36

## 4 Regression analysis

Regression analysis provides an effective framework for examining the influence of hybrid topological descriptors on the physicochemical behaviour of chemical compounds. We consider linear, quadratic and Exponential regression models to identify the one that best represents the relationship between topological indices and the empirical properties of PPI drugs.

$$\rho =\phi_{1}.\tau +\zeta$$ (1)

$$\rho =\phi_{1}\tau^{2}+\phi_{2}\tau +\zeta$$ (2)

$$\rho =\zeta e^{\phi_{1}\tau }$$ (3)

Here, ρ denotes the physicochemical property under consideration, τ represents the corresponding topological descriptor and $\phi_{i}$ and ζ are regression coefficients estimated from the observed data. The linear, quadratic and exponential models were obtained using the Curve Estimation procedure in SPSS. For the exponential model, the relationship was linearized through logarithmic transformation and fitted using the least-squares method. After conducting an extensive review and thorough examination of the physiochemical parameters outlined in Table 2, we evaluated each of the proposed indices (Tables 3 and 4). These indices correspond to the linear, quadratic and exponential regression models formulated in Equations 1–3 respectively.

**TABLE 3:** Topological indices of PPI drugs.

Drug	BM	TM	GH	GBM	GTM	HG
Omeprazole	276	478	153.3671	5.9255	3.4975	5.1506
Lansoprazole	284	506	157.7745	6.1131	3.5158	5.0359
Pantoprazole	291	501	160.6145	6.4209	3.8109	5.6161
Rabeprazole	272	458	148.4105	6.2772	3.8311	5.5395
Ilaprazole	314	538	173.3695	6.8654	4.0911	5.8425
Timoprazole	203	341	110.7013	4.6242	2.8113	3.8939
Tenatoprazole	261	451	144.3671	5.7255	3.3864	5.0395

**TABLE 4:** Topological indices of PPI drugs.

Drug	HBM	HTM	BMG	BMH	TMG	TMH
Omeprazole	1.2262	0.7457	115.0657	699	198.0102	1217
Lansoprazole	1.1639	0.6769	119.9307	716	213.7345	1284
Pantoprazole	1.3392	0.8171	123.0471	725	210.5236	1255
Rabeprazole	1.3276	0.8281	117.0165	662	195.2847	1125
Ilaprazole	1.3792	0.8381	132.0209	783	224.8172	1350
Timoprazole	0.9170	0.5653	86.9488	491	145.0727	831
Tenatoprazole	1.2039	0.7333	110.0657	654	189.0102	1136

The correlation coefficient and coefficient of determination criteria are presented in Equations 4, 5, respectively. A QSPR model is considered acceptable when the correlation coefficient r satisfies:

$$r=\frac{\sum_{i=1}^{N}\left(y_{A_{i}}-{\bar{y}}_{A}\right)\left(y_{P_{i}}-{\bar{y}}_{P}\right)}{\sqrt{\sum_{i=1}^{N}{\left(y_{A_{i}}-{\bar{y}}_{A}\right)}^{2}\;\sum_{i=1}^{N}{\left(y_{P_{i}}-{\bar{y}}_{P}\right)}^{2}}}\geq 0.7,$$ (4)

where $y_{A_{i}}$ denotes the experimental physicochemical property of the $i^{\text{th}}$ compound, ${\bar{y}}_{A}$ is the mean of the observed property values, $y_{P_{i}}$ represents the predicted value, ${\bar{y}}_{P}$ is the mean of predicted values and n is the total number of compounds.

A reliable and high quality predictive QSPR model should satisfy the following criterion:

$$R^{2}=1-\frac{\sum_{i=1}^{N}{\left(y_{A_{i}}-y_{P_{i}}\right)}^{2}}{\sum_{i=1}^{N}{\left(y_{A_{i}}-{\bar{y}}_{A}\right)}^{2}}>0.8.$$ (5)

Correlation values play a significant role in model development and are presented in Tables 5–7.

**TABLE 5:** Best performing topological indices (Linear model).

Property	Best predictor	r	$R^{2}$
BP	HTM	0.849	0.720
MR	HBM	0.944	0.891
MV	HG	0.945	0.893
MW	TMG	0.937	0.878
COM	TMH	0.986	0.972
POL	HBM	0.942	0.887
LogP	TMH	0.860	0.739

**TABLE 6:** Best performing topological indices (Quadratic model).

Property	Best predictor	r	$R^{2}$
BP	HBM	0.859	0.738
MR	HBM	0.949	0.900
MV	HG	0.986	0.972
MW	GBM	0.980	0.960
COM	TM	0.990	0.981
POL	HBM	0.946	0.896
LogP	TMG	0.895	0.802

**TABLE 7:** Best performing topological indices (Exponential model).

Property	Best predictor	r	$R^{2}$
BP	HTM	0.860	0.740
MR	HBM	0.949	0.899
MV	HG	0.939	0.882
MW	TMG	0.936	0.876
COM	TMH	0.971	0.942
POL	HG	0.946	0.896
LogP	TMG	0.901	0.811

### 4.1 Best-fit regression models and graphical representation

### 4.2 Regression analysis

Linear, quadratic and exponential regression analyses were performed to investigate the relationships between the calculated hybrid degree-based topological indices and the selected physicochemical properties of proton pump inhibitors. The statistical parameters of the optimal predictive models are presented in Table 8. The corresponding regression equations for the selected models are listed below:

$$\begin{array}{ll} BP & =397\;e^{0.529\left(HTM\right)}\\ MR & =-31.825+49.282\left(HBM\right)-39.214{\left(HBM\right)}^{2}\\ MV & =-481.480+251.970\left(HG\right)-21.325{\left(HG\right)}^{2}\\ MW & =-820.929+355.463\left(GBM\right)-26.485{\left(GBM\right)}^{2}\\ COM & =-453.586+3.070\left(TM\right)-0.002{\left(TM\right)}^{2}\\ POL & =-12.05+58.355\left(HBM\right)-15.239{\left(HBM\right)}^{2}\\ LogP & =0.134\;e^{0.014\left(TMG\right)} \end{array}$$

**TABLE 8:** Regression statistics of the optimal predictive models.

Property	Descriptor	Model	r	$R^{2}$	Adj. $R^{2}$	SE	Sig.
BP	HTM	Exp	0.860	0.740	0.688	0.034	0.013
MR	HBM	Quad	0.949	0.900	0.850	3.805	0.010
MV	HG	Quad	0.986	0.972	0.958	6.274	0.001
MW	GBM	Quad	0.980	0.960	0.939	10.259	0.002
COM	TM	Quad	0.990	0.981	0.971	10.816	0.000
POL	HBM	Quad	0.946	0.896	0.844	1.534	0.011
LogP	TMG	Exp	0.900	0.811	0.773	0.188	0.006

**FIGURE 2:**
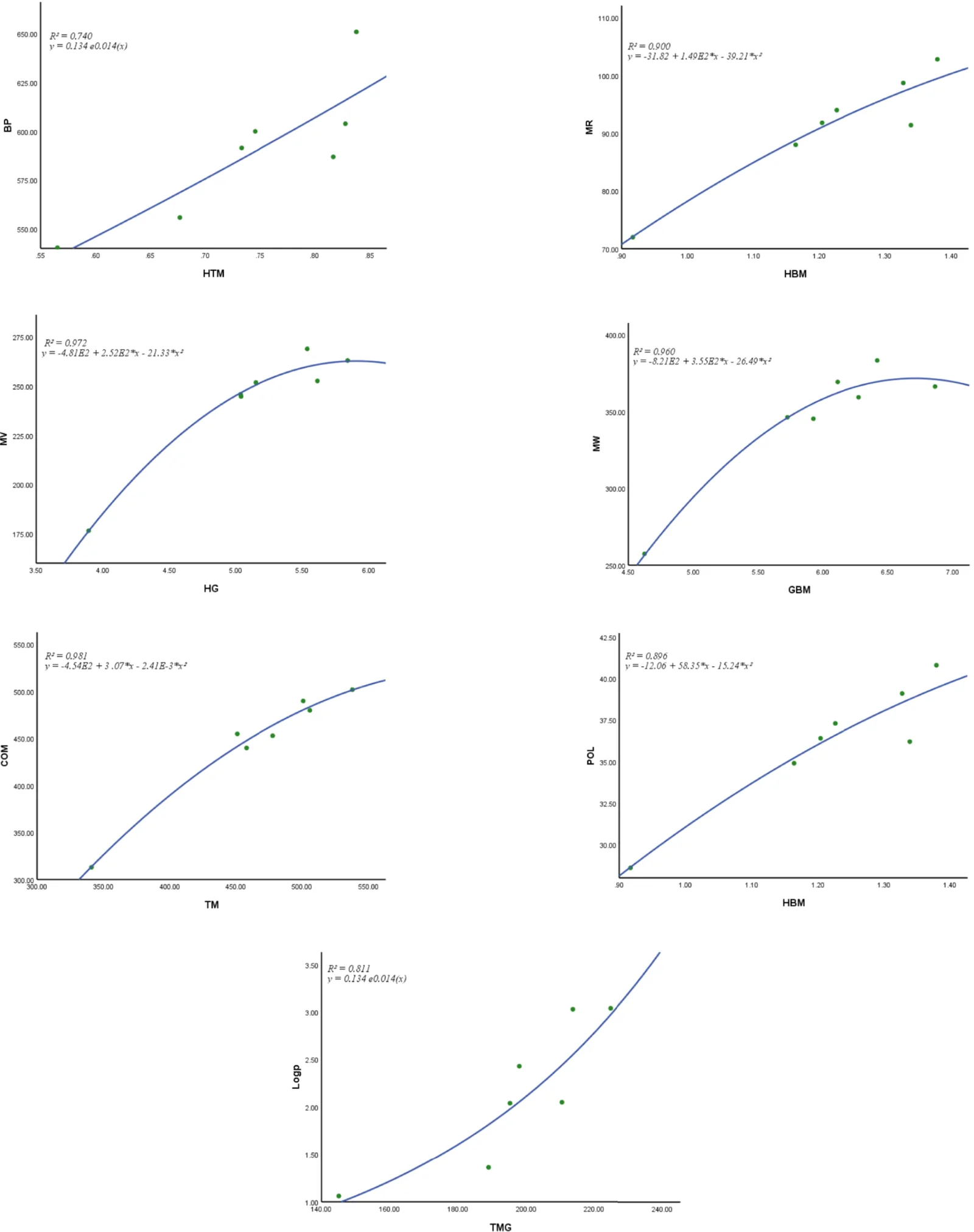
Graphical representation of best fit.

The obtained results demonstrate strong descriptor property relationships as evidenced by the high correlation coefficients and coefficients of determination observed for the regression models. A comparison of the three regression approaches revealed that the quadratic and exponential models generally provided superior fitting performance compared to the linear models. In particular, the exponential model was found to be most suitable for predicting boiling point and LogP, whereas quadratic models yielded the best performance for the remaining physicochemical properties. These findings highlight the effectiveness of the proposed hybrid topological indices in modeling and predicting the physicochemical behavior of proton pump inhibitors. The best fit regression plots corresponding to the selected descriptor property models are presented in Figure 2.

From Table 8, the best-performing topological indices for each property are summarized as follows:

- Boiling Point (BP) was predicted by the exponential model based on the HTM index yielding $R^{2}=0.740$ indicating a moderate predictive capability.
- Molar Refractivity (MR) shows an excellent fit with the HBM index with $R^{2}=0.900$ .
- Molar Volume (MV) is most accurately predicted by the HG index with a very high correlation $R^{2}=0.972$ .
- Molecular Weight (MW) demonstrates strong dependence on the GBM index yielding $R^{2}=0.960$ .
- Complexity (COM) is best modeled using the TM index with $R^{2}=0.981$ indicating a highly reliable prediction.
- Polarizability (POL) is effectively captured by the HBM index with $R^{2}=0.896$ .
- LogP was best predicted by the exponential model based on the TMG index yielding $R^{2}=0.811$ indicating a good predictive capability.

The experimental and predicted physicochemical properties of the PPI compounds obtained through the developed regression models are presented in Table 9. The close agreement between the observed and estimated values demonstrates the effectiveness and predictive capability of the proposed QSPR models.

**TABLE 9:** Experimental and predicted physicochemical properties of PPI compounds obtained using regression models.

PPI drug	BP		MR		MV		MW		COM		POL		LogP	
	Exp	Pre	Exp	Pre	Exp	Pre	Exp	Pre	Exp	Pre	Exp	Pre	Exp	Pre
Omeprazole	600	589	94	92.27	251.9	250.59	345.4	355.44	453	556.91	37.3	36.59	2.23	2.14
Lansoprazole	555.8	568	88	88.81	245.9	246.61	369.4	362.31	480	587.76	34.9	35.23	2.84	2.66
Pantoprazole	586.9	611	91.4	97.77	252.7	261.01	383.4	369.54	490	582.48	36.2	38.77	2.11	2.54
Rabeprazole	603.9	615	98.7	97.25	269	259.93	359.4	366.79	440	532.95	39.1	38.56	2.09	2.06
Ilaprazole	651	618	102.8	99.48	263.1	262.73	366.4	371.13	502	619.19	40.8	39.45	3.11	3.04
Timoprazole	540.3	535	72	72.10	176.5	176.33	257.3	256.47	313	360.72	28.6	28.65	1.02	1.06
Tenatoprazole	591.5	585	91.8	91.07	244.8	246.74	346.4	346.06	455	524.18	36.4	36.12	1.89	1.36

### 4.3 Validation

To assess the robustness and predictive ability of the developed QSPR models, internal and external validation techniques were employed. Leave-one-out cross-validation (LOOCV) was performed to evaluate the predictive stability of the models, while Y-randomization analysis was conducted to verify that the observed correlations were not obtained by chance. The corresponding validation statistics are presented in Table 10.

**TABLE 10:** Validation statistics of the developed QSPR models.

Property	Descriptor	Model	$R^{2}$	$Q_{\text{LOO}}^{2}$	Mean random $R^{2}$
BP	HTM	Exp	0.723	0.4713	0.157
MR	HBM	Quad	0.900	0.3560	0.345
MV	HG	Quad	0.972	0.6775	0.324
MW	GBM	Quad	0.960	0.5689	0.320
COM	TM	Quad	0.981	0.7507	0.336
POL	HBM	Quad	0.896	0.2890	0.331
LogP	TMG	Exp	0.786	0.6518	0.130

The obtained $Q_{\text{LOO}}^{2}$ values indicate acceptable predictive performance for most of the developed models with the highest value observed for COM $\left(Q_{\text{LOO}}^{2}=0.7507\right)$ . Furthermore, the mean randomized $R^{2}$ values were substantially lower than the corresponding original $R^{2}$ values, confirming that the descriptor property correlations are statistically meaningful and not the result of random associations. The high $R^{2}$ values obtained for all models demonstrate a strong agreement between the observed and predicted physicochemical properties. Moreover, all models yielded positive $Q_{\text{LOO}}^{2}$ values, indicating that they possess predictive capability beyond simple fitting of the available data. Although the $Q_{\text{LOO}}^{2}$ values varied among the developed models the results should be interpreted in the context of the limited number of clinically available proton pump inhibitors. Since the present study encompasses the entire set of seven PPI drugs considered the obtained validation statistics remain meaningful and provide valuable insight into the descriptor property correlations within this important class of compounds. Nevertheless, the combined results of regression analysis, LOOCV and Y-randomization results confirm the reliability and practical applicability of the developed QSPR models for the investigated physicochemical properties.

To examine the predictive ability of the developed QSPR models two structurally similar compounds, namely, Picoprazole and Linaprazan were included in the prediction analysis (Liu et al., 2024). The physicochemical properties of these compounds were estimated in Table 11 using the hybrid topological descriptors and the developed regression equations. The predicted results provide an additional assessment of the developed models on structurally related PPI derivatives that were not included in the model development stage. Although certain properties exhibit moderate deviations from the corresponding experimental values, the overall agreement between experimental and predicted values indicates that the proposed hybrid topological descriptors are capable of capturing the general structure property trends of PPI compounds.

**TABLE 11:** Experimental and predicted physicochemical properties of selected structurally related compounds using the developed quadratic QSPR models.

Drug	BP		MR		MV		MW		COM		POL		LogP	
	Exp	Pre	Exp	Pre	Exp	Pre	Exp	Pre	Exp	Pre	Exp	Pre	Exp	Pre
Picoprazole	597.1	580.1	92.4	91.10	243.1	249.14	343.4	356.04	490	546.32	36.6	36.13	1.96	2.13
Linaprazan	537.9	596	104.5	94.88	301	256.26	366.5	358.27	493	531.71	41.9	37.62	2.61	2.06

The intercorrelation matrix presented in Table 12 reveals the interrelationships among the hybrid topological indices for the PPI compounds.

**TABLE 12:** Intercorrelation matrix of the hybrid topological indices.

​	BM	TM	GH	GBM	GTM	HG	HBM	HTM	BMG	BMH	TMG	TMH
BM	1.0000	​	​	​	​	​	​	​	​	​	​	​
TM	0.9910	1.0000	​	​	​	​	​	​	​	​	​	​
GH	0.9990	0.9950	1.0000	​	​	​	​	​	​	​	​	​
GBM	0.9790	0.9500	0.9690	1.0000	​	​	​	​	​	​	​	​
GTM	0.9370	0.8860	0.9190	0.9860	1.0000	​	​	​	​	​	​	​
HG	0.9390	0.8940	0.9260	0.9790	0.9860	1.0000	​	​	​	​	​	​
HBM	0.9080	0.8540	0.8920	0.9560	0.9740	0.9960	1.0000	​	​	​	​	​
HTM	0.8350	0.7640	0.8140	0.9060	0.9480	0.9700	0.9880	1.0000	​	​	​	​
BMG	0.9970	0.9840	0.9930	0.9900	0.9550	0.9530	0.9220	0.8540	1.0000	​	​	​
BMH	0.9950	0.9970	0.9990	0.9560	0.9000	0.9110	0.8770	0.7950	0.9860	1.0000	​	​
TMG	0.9900	0.9980	0.9920	0.9580	0.8970	0.9000	0.8590	0.7700	0.9870	0.9910	1.0000	​
TMH	0.9800	0.9970	0.9880	0.9240	0.8510	0.8650	0.8240	0.7270	0.9670	0.9930	0.9910	1.0000

Since the intercorrelation matrix is symmetric, only the lower triangular entries are displayed; the corresponding upper triangular entries are omitted because they contain identical values.

## 5 Conclusion

In this study, a QSPR investigation of seven proton pump inhibitors, namely, Omeprazole, Lansoprazole, Pantoprazole, Rabeprazole, Ilaprazole, Timoprazole and Tenatoprazole was carried out using hybrid degree-based topological indices. The calculated descriptors were employed to model important physicochemical properties through linear, quadratic and exponential regression analyses. The obtained results revealed that several hybrid topological indices exhibit significant correlations with the investigated properties which leads to predictive models with satisfactory statistical performance. The quadratic and exponential models emerged as the most effective approaches for modeling the investigated physicochemical properties of proton pump inhibitors (PPIs). The results demonstrate the effectiveness of hybrid degree-based topological descriptors in capturing the structural characteristics of PPIs and predicting their physicochemical properties. Overall, the developed QSPR models provide valuable insight into this important class of drugs and establish a useful framework for future studies in molecular modelling, property prediction and drug design.

## Data Availability

The original contributions presented in the study are included in the article/supplementary material, further inquiries can be directed to the corresponding author.
